# Complex consequences of Cantu syndrome SUR2 variant R1154Q in genetically modified mice

**DOI:** 10.1172/jci.insight.145934

**Published:** 2021-03-08

**Authors:** Haixia Zhang, Alex Hanson, Tobias Scherf de Almeida, Christopher Emfinger, Conor McClenaghan, Theresa Harter, Zihan Yan, Paige E. Cooper, G. Schuyler Brown, Eric C. Arakel, Robert P. Mecham, Atilla Kovacs, Carmen M. Halabi, Blanche Schwappach, Maria S. Remedi, Colin G. Nichols

**Affiliations:** 1 Center for the Investigation of Membrane Excitability Diseases and; 2 Department of Cell Biology and Physiology, Washington University School of Medicine in St. Louis, St. Louis, Missouri, USA.; 3 Department of Molecular Biology, Center for Biochemistry and Molecular Cell Biology, Heart Research Center Göttingen, University Medicine Göttingen, Göttingen, Germany.; 4 DZHK (German Center for Cardiovascular Research), Partner Site Göttingen, Göttingen, Germany.; 5 Department of Medicine, Division of Endocrinology, Metabolism and Lipid Research,; 6 Department of Medicine, Division of Cardiology and; 7 Department of Pediatrics, Washington University School of Medicine in St. Louis, St. Louis, Missouri, USA.

**Keywords:** Muscle Biology, Vascular Biology, Cardiovascular disease, Genetic diseases, Potassium channels

## Abstract

Cantu syndrome (CS) is caused by gain-of-function (GOF) mutations in pore-forming (Kir6.1, *KCNJ8*) and accessory (SUR2, *ABCC9*) ATP-sensitive potassium (K_ATP_) channel subunits, the most common mutations being SUR2[R1154Q] and SUR2[R1154W], carried by approximately 30% of patients. We used CRISPR/Cas9 genome engineering to introduce the equivalent of the human SUR2[R1154Q] mutation into the mouse *ABCC9* gene. Along with minimal CS disease features, R1154Q cardiomyocytes and vascular smooth muscle showed much lower K_ATP_ current density and pinacidil activation than WT cells. Almost complete loss of SUR2-dependent protein and K_ATP_ in homozygous R1154Q ventricles revealed underlying diazoxide-sensitive SUR1-dependent K_ATP_ channel activity. Surprisingly, sequencing of SUR2 cDNA revealed 2 distinct transcripts, one encoding full-length SUR2 protein; and the other with an in-frame deletion of 93 bases (corresponding to 31 amino acids encoded by exon 28) that was present in approximately 40% and approximately 90% of transcripts from hetero- and homozygous R1154Q tissues, respectively. Recombinant expression of SUR2A protein lacking exon 28 resulted in nonfunctional channels. CS tissue from SUR2[R1154Q] mice and human induced pluripotent stem cell–derived (hiPSC-derived) cardiomyocytes showed only full-length SUR2 transcripts, although further studies will be required in order to fully test whether SUR2[R1154Q] or other CS mutations might result in aberrant splicing and variable expressivity of disease features in human CS.

## Graphical abstract

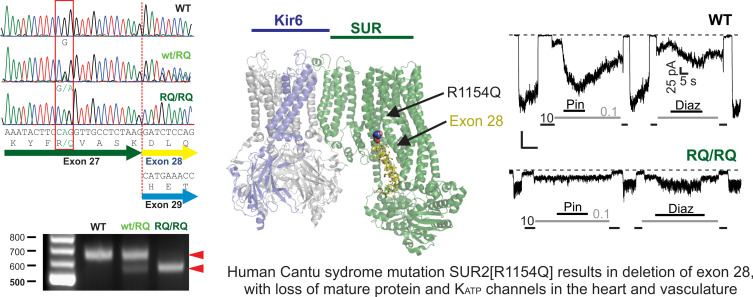

## Introduction

Cantu syndrome (CS), which has also been referred to as hypertrichosis-osteochondrodysplasia-cardiomegaly syndrome, is a rare multiorgan disease first recognized as such in 1982 (1). CS is characterized by congenital hypertrichosis, distinctive facial features, osteochondrodysplasia, and multiple cardiovascular abnormalities, including cardiomegaly, hypertrophy, pericardial effusion, pulmonary hypertension, and patent ductus arteriosus and cerebrovascular defects (2–5). Multiple reports have now confirmed that autosomal dominant gain-of-function (GOF) mutations in *KCNJ8* and *ABCC9*, the genes encoding the Kir6.1 and SUR2 subunits of ATP-sensitive potassium (K_ATP_) channels, represent the genetic basis of CS (6–11). The severity of features varies widely between individuals, although genotype-phenotype correlations have been difficult to establish; interestingly, patients with the same mutation can span the clinical spectrum (6, 7, 12, 13).

Expressed in various tissues in the body, K_ATP_ channels are nucleotide-gated, potassium selective channels that couple cellular metabolism to electrical excitability. *KCNJ8* and *ABCC9* are adjacent genes on human chromosome 12p12.1. A paralogous pair of genes (*KCNJ11* [Kir6.2] and *ABCC8* [SUR1]) is located on chromosome 11p15.1, with the result that multiple subunit combinations may exist in K_ATP_ channels in different tissues. K_ATP_ heterogeneity is further increased by the existence of variably spliced SUR isoforms (14–16); particularly prominent are 2 major splice isoforms of SUR2: SUR2A and SUR2B (17–19). K_ATP_ channels in vascular smooth muscle are predominantly composed of Kir6.1 and SUR2B (20, 21), whereas SUR2A predominates in cardiac ventricular myocyte K_ATP_ channels (22, 23).

To date, more than 30 missense mutations (24) have been identified in CS patients, and all mutations that have been functionally characterized result in GOF of K_ATP_ channels (7–11). In a previous study, we generated “CS mice,” in which the equivalent of human SUR2[A478V] and Kir6.1[V65M] mutations were introduced into the relevant mouse loci. In each case, key cardiovascular features of CS were replicated, and molecular, cellular, and systemic consequences increased from heterozygous (Het) to homozygous (Hom) conditions. Importantly, as the number of genetically confirmed CS cases has risen, it has become clear that residue R1154 in SUR2 is particularly susceptible to mutation, with 24 of 72 patients (20 of 57 unrelated families) carrying variants *ABCC9*[c.3461G>A], *ABCC9*[c.3460C>T], or *ABCC9*[c.3460C>G], which encode SUR2[p.R1154Q], SUR2[p.R1154W], and SUR2[p.R1154G], respectively (24). While this may reflect a susceptible genomic structure, at least the R1154Q mutation results in more severe GOF than many other CS mutations (7, 11), which raises the possibility that excessive occurrence of such mutations in patients reflects greater penetrance relative to other mutations. To gain further insight into the disease consequences of the R1154Q mutation, we used CRISPR/Cas9 genome engineering to generate CS mice carrying the SUR2[R1151Q] mutation, equivalent to human R1154Q. We show that the cardiovascular abnormalities in these CS mice were much less severe than in either of the 2 previously studied animal models, but this was accompanied by a marked decrease in SUR2-dependent K_ATP_ density, in both cardiac and smooth muscle cells. Further experiments showed that this was the result of the unanticipated appearance of *ABCC9* mRNA splicing, which led to in-frame exon deletion and loss of functional protein.

## Results

### The human R1154Q substitution causes K_ATP_ GOF, but only a mild CS phenotype.

CRISPR/Cas9 gene editing was used to introduce a single nucleotide mutation (*ABCC9*[c.3452G>A]; SUR2[p.R1151Q]) in the endogenous mouse *ABCC9* locus, resulting in protein substitution analogous to the most common human CS mutation, SUR2[R1154Q]. Both heterozygous (SUR2^WT/RQ^) and homozygous (SUR2^RQ/RQ^) mice were viable and fertile. We subsequently analyzed cellular, organ, and whole animal phenotypes of these animals, which we refer here to as SUR2[R1154Q] mice to distinguish it from human CS.

**Figure 1:**
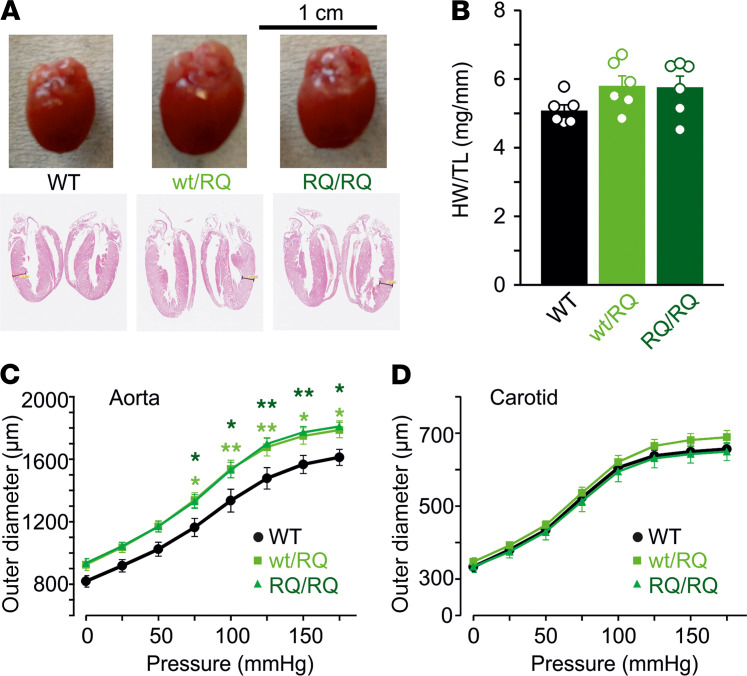
Cardiovascular phenotype of R1154Q mice. (**A** and **B**) Cardiomegaly in heterozygous SUR2^WT/RQ^ (WT/RQ) and homozygous SUR2^RQ/RQ^ (RQ/RQ) hearts. (**C** and **D**) Isolated ascending aortas of WT/RQ and RQ/RQ hearts show similar increases in diameter at all pressures relative to WT (**C**), but carotid artery mechanical properties are not different from those of WT (**D**) (*n* = 5 for WT, *n* = 7 for WT/RQ, *n* = 6 for RQ/RQ). Statistical significance was determined by 2-way ANOVA followed by post hoc Tukey’s test correction for multiple comparisons;*P < 0.05, **P < 0.01 compared with WT. HW, heart weight; TL, tibia length.

**Figure 2:**
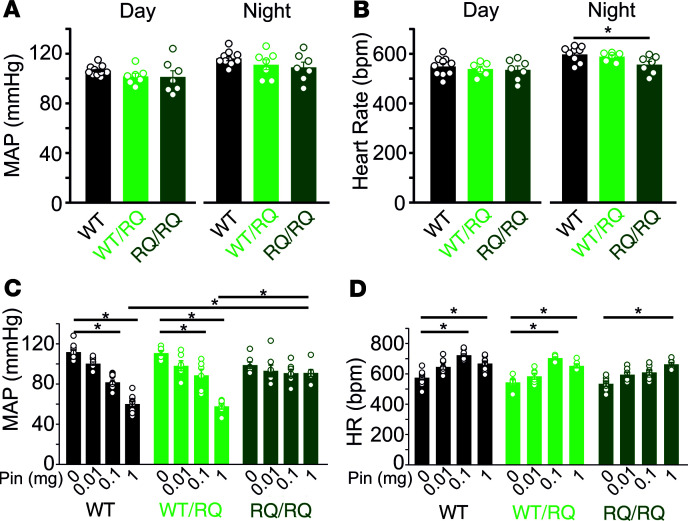
Cardiovascular function of R1154Q mice. (**A**) Mean arterial pressure (MAP) and (**B**) mean HR in conscious WT, heterozygous SUR2^WT/RQ^, and SUR2^RQ/RQ^ mice during day and night. (**C**) MAP and (**D**) HR in anesthetized mice showing blunted response to the K_ATP_ channel activator pinacidil (Pin) in SUR2^RQ/RQ^ mice. Statistical significance was determined by 1-way ANOVA followed by Tukey’s test for pairwise comparison; asterisks indicates significant difference (*P* < 0.05) within genotypes.

One of the most consistent features of patients with CS is pronounced cardiomegaly (24, 25). Consistent with this, hearts were larger in heterozygous SUR2^WT/RQ^ than WT mice, but not obviously more so in homozygous SUR2^RQ/RQ^ mice (Figure 1, A and B). Therefore, R1154Q hearts displayed chamber dilation and cardiac enlargement similar to, although much less dramatic than, that seen previously in A478V or V65M CS mouse hearts (26). Isolated aortic diameter was greater in SUR2^WT/RQ^ than WT mice at all pressures (Figure 1C) although, again, there was no further increase in SUR2^RQ/RQ^ mice, and carotid artery dimensions were not different between WT and R1154Q animals (Figure 1D). Slope compliance (reflecting noncontractile biomechanical properties) was not obviously different between genotypes (Figure 1C). As shown in Figure 2A, both SUR2^WT/RQ^ and SUR2^RQ/RQ^ mice maintained diurnal fluctuation in blood pressure, but unlike in SUR2[A478V]-expressing mice (26), blood pressures were not significantly lower than control in either SUR2^WT/RQ^ or SUR2^RQ/RQ^ mice (Figure 2A). Moreover, while pinacidil had similar BP-lowering effect in control and Het SUR2^WT/RQ^ mice, it had almost no effect on BP in SUR2^RQ/RQ^ mice (Figure 2C). In contrast, pinacidil raised heart rates (HRs) similarly in all genotypes (Figure 2D).

### Unexpected K_ATP_ channel properties in R1154Q cardiac and vascular smooth muscle cells.

**Figure 3:**
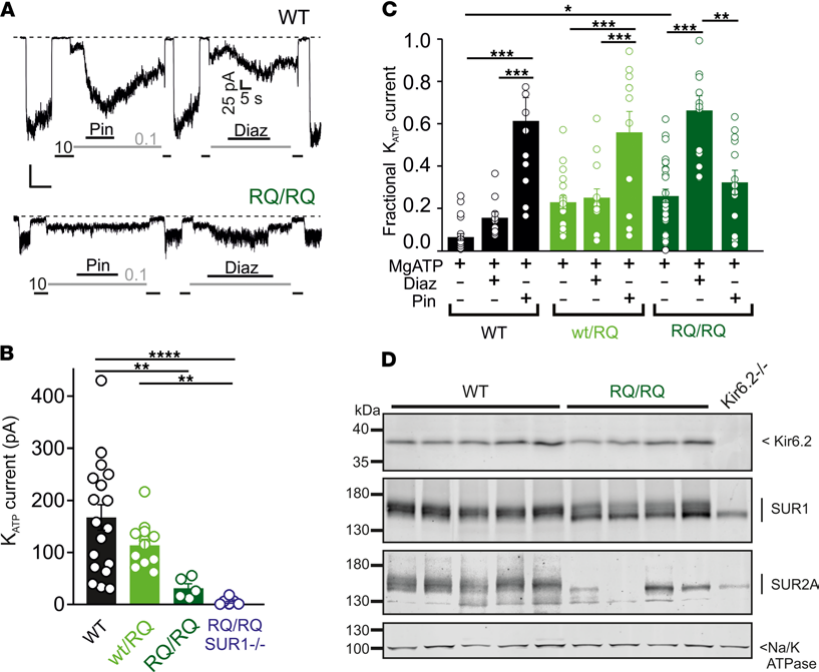
Decreased K_ATP_ channel density and switch to SUR1 dependence in SUR2A[R1154Q] hearts. (**A**) Representative inside-out patch clamp recordings of K_ATP_ channel activity from acutely dissociated ventricular myocytes from WT and SUR2^RQ/RQ^ mice. Inhibition by 10 or 0.1 μM MgATP and the response to the K channel openers pinacidil and diazoxide (Diaz) at 100 μM, in the presence of MgATP (recording at –50 mV membrane potential), are shown. (**B**) Absolute K_ATP_ current level in zero ATP, from experiments as in **A**. (**C**) K_ATP_ current, as a fraction of current in zero ATP, from experiments as in **A**. (**D**) Western blot analysis of the membrane fraction from ventricular heart tissue of WT and SUR2^RQ/RQ^ mice (4 biological replicates each) showing protein steady-state levels of K_ATP_ channel subunits and Na/K-ATPase α subunits. Since both SUR subunits are only core-glycosylated when the Kir6.2 subunit is missing (28), tissue from a single Kir6.2^–/–^ mouse is also shown for reference. Statistical significance was determined by 1-way ANOVA followed by Tukey’s test for pairwise comparison; * *P* < 0.05, ** *P* < 0.01, *** *P* < 0.001.

Mice expressing introduced SUR2[A478V] and Kir6.1[V65M] CS GOF mutations exhibit marked lowering of blood pressure and cardiac enlargement (26). Since previous studies show that recombinant SUR2[R1154Q] causes a significant GOF — in both human SUR2 (7) and rat SUR2 with the identical DNA mutation (11) — the above results (i.e., limited or no increase in vessel diameters and compliance, lack of effect on BP, and lack of pinacidil action in SUR2^RQ/RQ^) are unexpected, and raise questions regarding the level and nature of K_ATP_ channels in these mutant tissues. We therefore examined the density of K_ATP_ channels, and sensitivity to the K_ATP_ channel openers pinacidil (acting primarily on SUR2) and diazoxide (acting primarily on SUR1) in excised membrane patches from ventricular myocytes (Figure 3A). Overall K_ATP_ channel density was much lower than WT in SUR2^WT/RQ^ myocytes, and dramatically so in homozygous SUR2^RQ/RQ^ myocytes (Figure 3B). Moreover, in striking contrast to the findings in recombinant R1154Q channels, pinacidil-mediated activation was essentially absent in homozygous SUR2^RQ/RQ^ cardiomyocytes, while relative diazoxide-mediated activation was markedly enhanced (Figure 3C).

**Figure 4:**
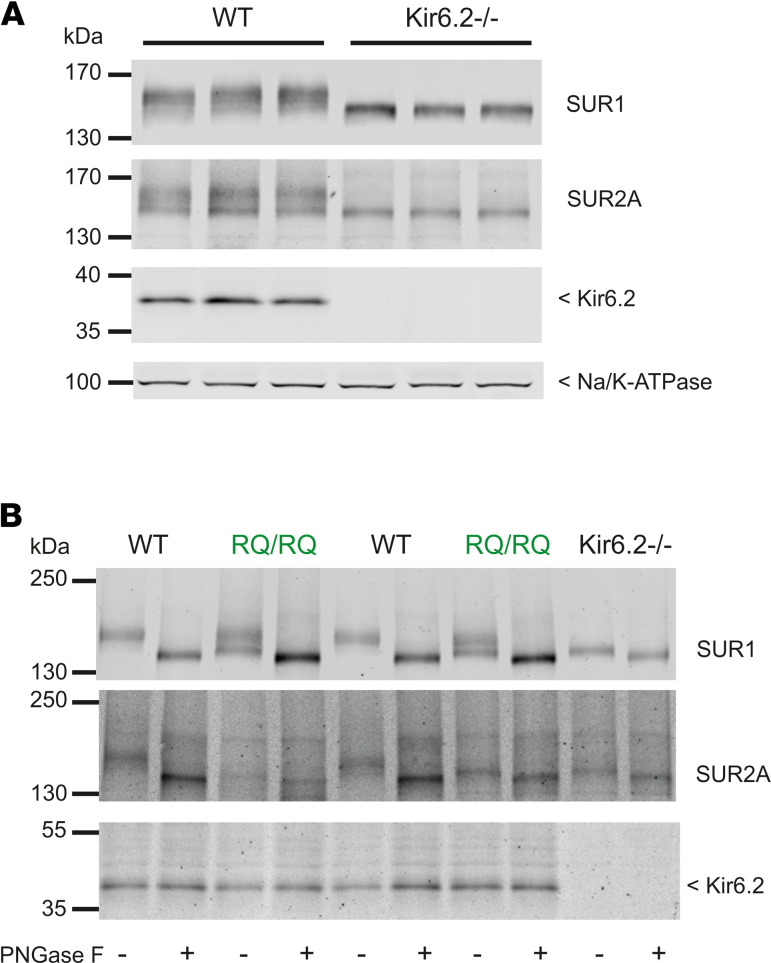
SUR1 and SUR2A maturation in SUR2A[R1154Q] hearts. (**A**) Western blot analysis of the membrane fraction from ventricular heart tissue of WT and Kir6.2^–/–^ mice. Representative result from 3 biological replicates. (**B**) PNGase F treatment (18.75 U/μL) of ventricular heart membrane lysates of WT, SUR2^WT/RQ^, SUR2^RQ/RQ^, and Kir6.2^–/–^ mice. Representative Western blot of 2 biological replicates.

This unexpected lowering of channel density and apparent switch in pharmacological sensitivity from pinacidil to diazoxide suggests that levels of SUR2-dependent channel complexes are reduced in R1154Q hearts, almost completely in homozygous SUR2^RQ/RQ^ hearts, and that the remaining functional sarcolemmal channels are predominantly SUR1 dependent. To test the latter suggestion directly, we additionally generated SUR2^RQ/RQ^ mice on a SUR1^–/–^ background (27); in this case, no K_ATP_ was detected (Figure 3B), indicating that channels in SUR2^RQ/RQ^ animals are essentially SUR1 dependent. We further examined underlying K_ATP_ subunit levels in isolated ventricular tissue by Western blot analysis (Figure 3D). This revealed a marked decrease in core- and complex-glycosylated SUR2A proteins in homozygous SUR2^RQ/RQ^ hearts compared with littermate control hearts (Figure 3D). In contrast, levels of core-glycosylated SUR1 protein were increased in SUR2^RQ/RQ^ hearts (Figure 3D). Both SUR1 and SUR2A in WT hearts are normally associated with Kir6.2 (28), but only core-glycosylated, approximately 140 kDa forms of both SUR1 and SUR2A were present in Kir6.2^–/–^ hearts (Figure 4A), demonstrating that they both require association specifically with Kir6.2 to mature. As shown in Figure 4B, treatment with peptide:N-glycosidase F (PNGase F) resulted in complete deglycosylation of both SUR1 and SUR2A, demonstrating that total SUR1 was indeed increased in SUR2^RQ/RQ^ hearts, although the excess was incompletely glycosylated. The results confirm that, consistent with loss of SUR2A-dependent K_ATP_ current and relative increase in SUR1-dependent channels, mature SUR2A protein levels were reduced, while total SUR1 protein was increased, in SUR2^RQ/RQ^ hearts.

**Figure 5:**
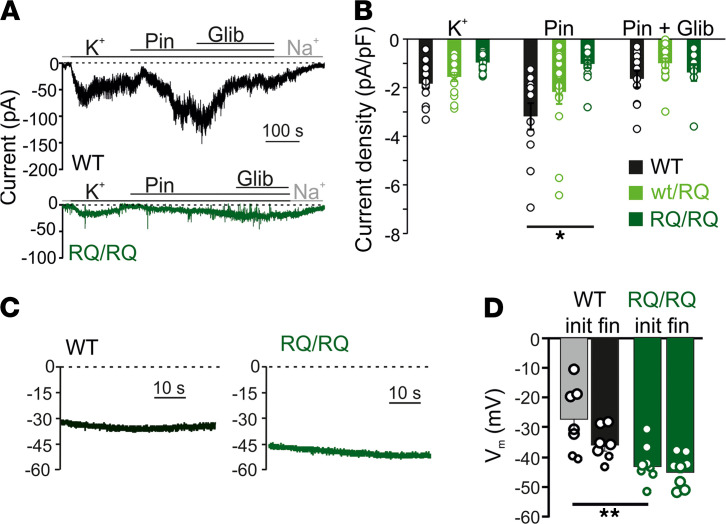
Loss of pinacidil-sensitive SUR2-dependent K_ATP_ channels in SUR2A[R1154Q] vascular smooth muscle. (**A**) Representative whole-cell voltage clamp recordings from acutely isolated aortic smooth muscle cells from WT and SUR2^RQ/RQ^ mice. Cells were voltage clamped at –70 mV. Glib, glibenclamide. (**B**) Summary of whole-cell current densities from voltage clamp recordings as in **A**, showing significantly reduced pinacidil-activated K_ATP_ conductance in SUR2^RQ/RQ^ cells. Statistical significance was determined by multi-way ANOVA, followed by 2-tailed *t* test pairwise comparison with Bonferroni’s correction for multiple comparisons (adjusted α = 0.008); *P < 0.008. (**C**) Representative whole cell current-clamp recordings from acutely isolated aortic smooth muscle cells from WT and homozygous SUR2^RQ/RQ^ mice using an intracellular pipette solution absent of nucleotides. (**D**) Summary of initial (init) and final (fin) membrane potentials from experiments as in **C**. Statistical significance was determined by 1-way ANOVA followed by Tukey’s tests; ** *P* < 0.01.

The effects of the introduced mutation on K_ATP_ channel function were also examined in vascular smooth muscle cells (VSMCs). In contrast to the findings in A478V and V65M animals (26), whole-cell patch clamp recordings using an intracellular pipette solution containing no ATP (see Methods) revealed no elevation of basal K_ATP_ conductance in acutely isolated aortic smooth muscle cells from SUR2^WT/RQ^ compared with WT mice, and significantly lower conductance in SUR2^RQ/RQ^ compared with WT cells (Figure 5, A and B). Application of pinacidil provoked a significant increase in conductance in WT VSMCs, but there was less of an effect in SUR2^RQ/RQ^ and very little effect in VSMCs from SUR2^RQ/RQ^ mice (Figure 5, A and B). These results indicate that K_ATP_ density was also markedly reduced in R1154Q smooth muscle, although SUR2^RQ/RQ^ VSMCs were hyperpolarized relative to WT VSMCs following break-in in current clamp mode (Figure 5, C and D), indicating at least some net K_ATP_ GOF under intact cell physiological conditions in SUR2^RQ/RQ^ VSMCs.

Taken together, the data indicate that, while the expected molecular consequence of the SUR2[R1154Q] substitution is a significant GOF of SUR2-dependent K_ATP_ channels in blood vessels and the heart, there were minimal cardiovascular CS features. There was an unexpected downregulation of SUR2-dependent K_ATP_ channel density in heterozygous SUR2^WT/RQ^ cardiac and vascular smooth muscle myocytes — dramatically so in homozygous SUR2^RQ/RQ^ tissues — accompanied by an increase in SUR1 levels in the heart.

### Unanticipated alternate splicing of SUR2 exon 28 in R1154Q tissues.

**Figure 6:**
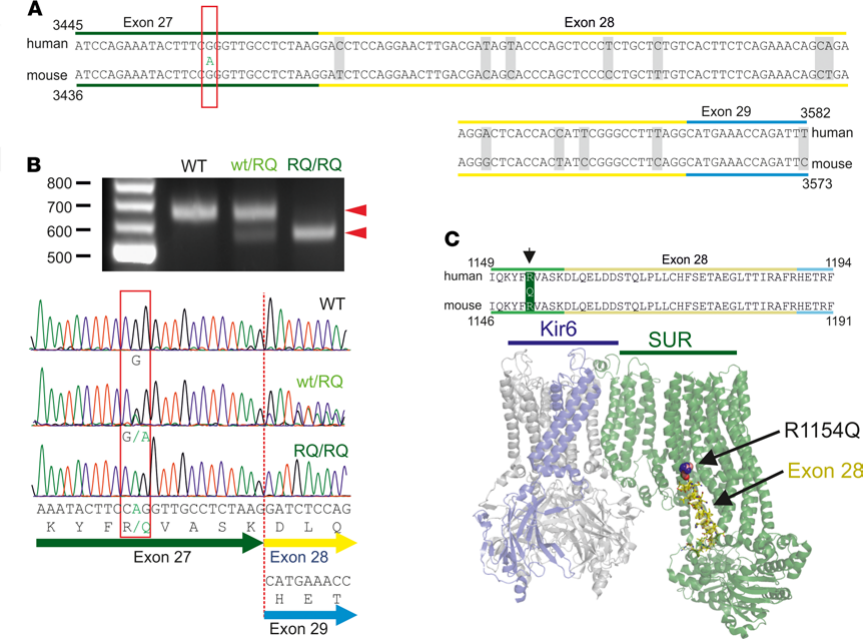
Abnormal splicing in SUR2A[R1154Q] mRNA. (**A**) Canonical cDNA sequence for human and mouse SUR2 over the exon 27–29 region (non-identities indicated by gray). Human nucleotide c.3461 (mouse c.3452) G>A mutation generating p.R1154Q is indicated by the red box. (**B**) Analysis of cDNA PCR product by gel electrophoresis and by direct sequencing of the selected bands reveals an exact deletion corresponding to the 93 nucleotides of exon 28 in approximately half of heterozygous WT/RQ and almost all homozygous RQ/RQ mouse transcripts (red arrowheads). (**C**) Top: Amino acid sequence of residues 1149–1194 (human) is identical in human and mouse SUR2. Bottom: Model of the Kir6/SUR complex (Protein Data Bank 5WUA) indicates the predicted location of the R1154Q mutation and amino acids encoded by exon 28.

The above results led us to conclude that although the R1154Q mutation indeed causes a GOF in K_ATP_ channel properties (since smooth muscle is still relatively hyperpolarized), the expressivity of CS features is severely blunted in these animals by the unexpected reduction in SUR2-dependent K_ATP_ density that is not seen in other (A478V, V65M) CS mice (26). In homozygous SUR2^RQ/RQ^ mice there was almost complete disappearance of SUR2-dependent K_ATP_ channels in both heart and blood vessels, and a consequent reduction in disease severity, as reflected by lack of obvious effects on BP and reduced effects on heart size (Figures 1 and 2). We considered the possibility that CRISPR-generated mistakes may have led to additional mutations that resulted in defective protein, but sequencing of gDNA more than 5000 bp on either side of the introduced mutation failed to detect any additional mutations (data not shown). It has long been recognized that there are multiply spliced forms of the SUR2 protein (15, 29–31), the best characterized being the SUR2A and SUR2B isoforms, which result from alternate splicing of the terminal exon 38A/B. The R1154Q and R1154W mutations are in exon 27, and while there is to our knowledge no evidence in the literature for alternate splicing of this region of the gene, the specific location of the underlying mutations (13 and 14 bases, respectively, before the end of exon 27; Figure 6, A–C) places them in a potential exon splicing enhancer (ESE) region that may influence exon splicing (32). We isolated mRNA from WT and R1154Q mouse hearts, generated cDNA corresponding to SUR2A and SUR2B, and sequenced the entire coding region. The introduced c.3452G>A mutation was present in fewer than 50% of heterozygous SUR2^WT/RQ^ and close to 100% of homozygous SUR2^RQ/RQ^ transcripts, but, strikingly, heterozygous cDNA reads became doubled sequences immediately following the last nucleotide of exon 27 (Figure 6B). Close inspection revealed that this corresponds to approximately half of the reads in heterozygous SUR2^WT/RQ^, and essentially all reads in homozygous SUR2^RQ/RQ^, transcripts, reflecting an exact in-frame deletion of the 93 bases in the following exon, exon 28 (Figure 6B).

It might be hypothesized that this exon 28 splicing of SUR2 mRNA could be a cellular regulatory mechanism to moderate abnormally increased K_ATP_ channel function activity. However, this does not seem likely, since no alternative splicing of exon 28 was detected in heterozygous or homozygous SUR2[A478V] or Kir6.1[V65M] hearts (data not shown), or in WT hearts (Figure 6B). Instead, the tight dependence of splicing on the presence of the c.3452G>A mutation indicates that the nucleotide change itself is directly responsible for the splicing event.

**Figure 7:**
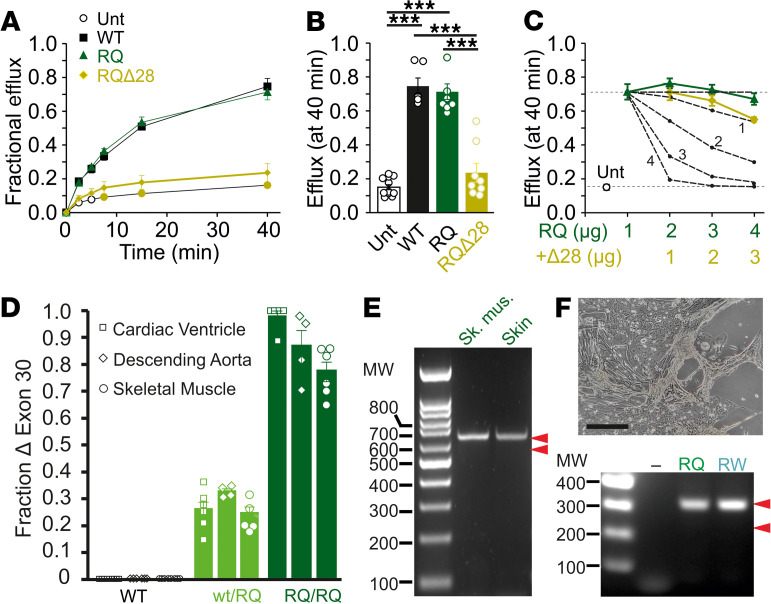
Functional consequence of SUR2[R1154Q] mRNA splicing. (**A**) Representative Rb efflux experiments from untransfected COSm6 cells (Unt) and cells transfected with WT, R1154Q (RQ), and R1154Q[Δexon28] SUR2A, plus Kir6.2. (**B**) Fractional efflux at 40 minutes, from experiments as in **A**. Statistical significance was determined by 1-way ANOVA followed by Tukey’s tests; ***P < 0.001. (**C**) Fractional efflux at 40 minutes, from experiments similar to those in **A**, in cells transfected with SUR2[R1154Q,Δexon28] subunits in addition to SUR2[R1154Q] (plus Kir6.2) subunits. Dashed lines are predicted levels of efflux assuming that 1, 2, 3, or 4 WT subunits in a randomly assembling complex are necessary to restore function. (**D**) cDNA PCR product analyzed by gel electrophoresis reveals similar levels of splicing in R1154Q ventricle, smooth muscle, and skeletal muscles, suggesting that SUR2 function will be significantly reduced in all tissues. (**E**) cDNA PCR product from human R1154Q patient skeletal muscle and skin analyzed by gel electrophoresis reveals only a single band corresponding to a 642 bp fragment from full-length SUR2A cDNA and no band corresponding to the predicted 549 bp from exon 28–deleted cDNA (red arrowheads). (**F**) Image of R1154Q patient iPSC–derived cardiomyocytes (scale bar: 50 μm). cDNA PCR product from R1154Q or R1154W patient iPSC–derived cardiomyocytes analyzed by gel electrophoresis reveals only a single band corresponding to the 325 bp fragment from full-length SUR2A cDNA and no band corresponding to the predicted 232 bp fragment from exon 28–deleted cDNA (red arrowheads) (representative result from *n*= 3 repeats).

To assess the effect of deleting exon 28 on K_ATP_ channel activity, we engineered SUR2A cDNA with exon 28 deleted. When coexpressed with WT Kir6.2, SUR2A[R1154Q,Δexon28] failed to generate significant K_ATP_ channel activity in heterologous expression (Figure 7, A and B). In subunit mixing experiments, with equal transfection of WT SUR2A and SUR2A[R1154Q,Δexon28] cDNA, there was no evidence for dominant-negative suppression of heterologously expressed K_ATP_ channels by exon-deleted subunits (Figure 7C). The data were best fit under the assumption that even 1 full-length WT subunit would be sufficient to rescue function (Figure 7C), consistent with truncated subunits being rapidly degraded and not incorporated into K_ATP_ complexes.

### Lack of splicing in human R1154Q mutant tissues or induced pluripotent stem cell–derived cells.

The introduced mutation thus results in alternate splicing and consequent loss of SUR2 protein in CS mice. In turn, this leads to significantly blunted phenotype severity, despite the R1154Q mutation showing a marked molecular GOF (11).If the same splicing is similarly present in humans, it would tend to mitigate the effects of the mutation. In addition, variable SUR2 splicing between individuals could potentially account for the quite variable expressivity in CS individuals with the R1154Q mutation (24). We further examined the tissue dependence of exon 28 skipping in cDNAs generated from mRNA isolated from multiple R1154Q mouse tissues. As shown in Figure 7D, the apparent fraction of spliced transcripts was similar in skeletal, smooth, and cardiac muscle, being approximately 25%–35% in heterozygous, SUR2^WT/RQ^ and approximately 75%–100% in homozygous, SUR2^RQ/RQ^ animals. This further indicates that alternate SUR2 splicing is driven by the nucleotide change via a cell-autonomous mechanism, independent of tissue type. The less-than-stoichiometric ratio of spliced to unspliced transcript in the heterozygous case further suggests slower transcription or reduced stability of the mutant mRNA. We obtained a skin and skeletal muscle biopsy sample from a single R1154Q patient and successfully isolated *ABCC9* mRNA. However, PCR from both samples revealed only single product bands corresponding to full-length SUR2A cDNA and no band corresponding to exon 28–deleted cDNA (Figure 7E).

We also obtained PBMCs from a patient with the R1154Q mutation, and renal epithelial cells (RECs) were obtained from a patient with the R1154W mutation. Human induced pluripotent stem cells (hiPSCs) were generated from these primary cells using Sendai virus–based reprogramming vectors. Two subclonal hiPSC lines were produced for each mutation, and DNA sequencing analysis confirmed the expected mutation in each CS hiPSC line. A GCaMP6-expressing hiPSC line from an unaffected individual was used as a control. Expression of human pluripotency-associated genes and a normal karyotype were confirmed for all hiPSCs prior to subsequent experiments. WT and CS hiPSCs were differentiated into cardiomyocytes as previously described (33). When we used this approach, hiPSC-derived cardiomyocytes exhibiting robust rhythmic contractile behavior were present by days 7–9. Subsequently, a 10-day lactate purification step was used to metabolically select for cells (Figure 7F) with cardiomyocyte-specific biochemical properties enabling survival exclusively via lactate metabolism, as previously described (34). For unknown reasons, we were unable to detect K_ATP_ channels in these myocytes (data not shown). However, RT-PCR analysis of RNA isolated from WT, R1154Q, and R1154W hiPSC–derived cardiomyocytes on day 45 revealed full-length SUR2A transcripts in each genotype, with no evidence of detectable alternate splicing (Figure 7F). Although the amino acid sequence in this region of SUR2 is identical in mice and humans (Figure 6C), there are slight variations in codon usage between the 2 species (Figure 6A) that could affect *ABCC9* mRNA splicing. Nevertheless, the lack of detectable alternate splicing in human R1154Q and R1154W iPSC–derived cardiomyocytes suggests that the disease mutation may not lead to alternate splicing in native human tissues.

## Discussion

### The cellular pathology of K_ATP_ GOF in Cantu syndrome: additional outcome twists with R1154Q.

The present study demonstrates that, when introduced into the mouse genome, the most common CS-associated *ABCC9* mutation (encoding human SUR2[R1154Q]) resulted in qualitatively the same cardiovascular features as the SUR2[A478V] and Kir6.1[V65M] mutations (26), providing further confirmation of the common cardiovascular outcome of vascular dilation and cardiac enlargement resulting from SUR2- or Kir6.1-dependent K_ATP_ GOF in CS. However, in SUR2[R1154Q] animals, the disease was quantitatively much less severe than naively predicted based on the molecular severity of the mutation. The reason for the reduced severity of outcome was shown to be a reduction in overall K_ATP_ density in both vascular smooth muscle and heart, particularly in the case of homozygous SUR2^RQ/RQ^ mice. This in turn was shown to be a result of the genomic c.3452G>A mutation causing altered pre-mRNA splicing, with deletion of the following exon 28 and generation of nonfunctional SUR2 proteins, and downregulation of overall K_ATP_ density. SUR2A levels were strongly reduced in homozygous SUR2^RQ/RQ^ mice, with the SUR2A protein that was present showing lower complex glycosylation, indicative of ER localization, as seen in Kir6.2-knockout animals (28).

In general, genetic or pharmacological manipulations that alter the levels of any K_ATP_ subunits, even complete knockout of any given subunit, have not been shown to result in marked compensatory changes in other subunits, in any tissues (35–37). In the present case, there was a surprising increase in relative diazoxide sensitivity of cardiac K_ATP_, accompanied by a small increase in absolute levels of diazoxide-sensitive current, and of mature, glycosylated SUR1 protein, in the heart. Previous studies have suggested that SUR1, while present in the heart, loses out to SUR2A in competition for association with Kir6 subunits, resulting in low levels of fully mature, glycosylated SUR1 being present in K_ATP_ channels at the membrane surface (28). We speculate this may be because SUR2A-containing channels normally leave the secretory pathway more efficiently, so that when mutant SUR2A protein is depleted, SUR1 accesses Kir6.2, which explains the higher levels of core-glycosylated SUR1 and diazoxide-activatable current in R1154Q hearts.

Cardiac hypertrophy and enhanced cardiac output are a consistent finding in CS patients (25) and in both Kir6.1[V65M] and SUR2[A478V] mutant mice (26). As we have shown in these mice, cardiac hypertrophy arises independently of ventricular K_ATP_ activity, as a secondary consequence of enhanced vascular K_ATP_ activity, resulting in vasodilation; reduced vascular resistance; and, in response, enhancement of renin-angiotensin signaling, adrenergic signaling, or other vasoresponsive pathways (26, 38). We also saw cardiac enlargement in SUR2[R1154Q] mutant mice, but this was less marked than in V65M or A478V mice, consistent with reduced overall expression of SUR2-dependent K_ATP_ channel levels in the vasculature (as well as the heart). Cardiac β-adrenergic receptor (β-AR) activation promotes cardiac hypertrophy (39) and, as we have also shown, can enhance trafficking of SUR1-dependent K_ATP_ channels to the myocardial surface membrane (28). This in turn could contribute to increased SUR1-containing channels at the cell surface in R1154Q hearts.

### Variable disease-causing/modifying consequences of alternate splicing in ABCC genes.

Strikingly, the SUR2[R1154Q] (*ABCC9* c.3461G>A) mutation induced alternate splicing of SUR2 mRNA, generating a truncated SUR2 protein that lacks the 30 amino acids of exon 28. When expressed together with Kir6.2 in recombinant cells, the SUR2[R1154Q,Δexon28] construct failed to generate active K_ATP_ channels. In mixed expression with full-length SUR2A cDNA, there was no evidence for a dominant-negative effect of the SUR2[R1154Q,Δexon28] construct; the data were best fit by assuming that even 1 full-length subunit was sufficient to rescue function. This can explain the observed reduction in overall channel density yet persistence of vascular hyperpolarization and cardiac enlargement in R1154Q animals; in the case of heterozygous mice, the disease features were less marked than seen in heterozygous SUR2[A478V] animals (26, 38), even though the molecular consequence of the mutation itself was more severe (7, 11). In contrast to SUR2[A478V] animals, the disease features of homozygous SUR2[R1154Q] animals were no more dramatic than those of the heterozygous animals, and K_ATP_ channel activities were more markedly decreased, particularly in smooth muscle — explained by the enhanced degree of splicing.

In many genes, exon inclusion/skipping is becoming recognized as a more common consequence of disease mutations than previously assumed (40–42). Multiple intra-intronic and intra-exonic mutations in CFTR (*ABCC7*), a gene closely related to *ABCC8*, have been associated with nonfunctional protein and cystic fibrosis (CF) disease (43). In one systematic study involving both in silico predictions and analysis of exon skipping in recombinant minigenes (44), 9 of 19 disease-associated CFTR mutations induced exon skipping in a fraction of transcripts, but did not abolish WT expression completely, potentially underlying variably milder phenotypes. Mutations occurring at conserved intron–exon boundaries (i.e., splicing junctions at the −1, −2, −3, and +1, +2, +3 positions) are expected to affect splicing of the immediately adjacent exons. The consequence of such mutations, for example, c.1117-1G>A and c.1209+1G>A in *ABCC7*, are generally considered to be severe, whereas mutations occurring at more distant positions, for example, +5, +6, or −5 and −6, are mild, typically associated with only mild CF disease (45).

Alternate splicing is well recognized as a component of *ABCC9* regulation; the canonical finding is that cardiomyocytes express SUR2A, a variant containing exon 38A, whereas smooth muscle cells typically express SUR2B, containing the alternate C-terminal exon 38B. Previous studies in mice have also identified multiple additional potential spliced SUR2 variants (14, 15, 19), including short forms of only 28 and 68 kDa (29), in addition to the full-length (~150 kDa) form in the WT cardiac sarcolemmal membrane. Some small exon deletions modulate channel ATP sensitivity (15), whereas coimmunoprecipitation of short forms lacking NBD1 but containing NBD2 with Kir6.1 or Kir6.2 suggests that abnormal channel properties could be generated (29). Other studies identified an additional 55 kDa form of the protein lacking exons 5–28 in mitochondria (termed mitoSUR2) generated by a nonconventional intraexonic splicing (IES) event within the 4th and 29th exons of SUR2 mRNA (46). Specific deletion of exon 5 of *ABCC9*, to ablate expression of both plasma membrane and the mitoSUR2 short form, resulted in neonatal cardiomyopathy, potentially due to failure of the heart to transition normally from fetal to mature myocardial metabolism (47). Conversely, mice overexpressing the 55 kDa short-form protein had improved recovery from ischemia/reperfusion injury relative to WT hearts (48).

Such studies indicate that exon splicing could result in distinct forms of the protein that are expressed in different cellular compartments, with profoundly different effects on cell function. The present data raise the possibility that R1154Q (or R1154W, and perhaps other) CS mutations might result not only in a functional K_ATP_ GOF but, by causing exon skipping, but also in a truncated protein and hence an effective *mixed* loss/GOF phenotype, potentially explaining the variable expressivity of disease features in human CS (24). In cardiomyocytes derived from R1154Q CS patient iPSCs, we failed to detect any exon 28 skipping. The amino acid sequence in the region of R1154 is identical in mouse and human, but there is some variation in codon usage between the 2 species, and, although splicing prediction algorithms suggest that the human mutation and the CRISPR-introduced mouse mutation should alter exon splicing similarly, it is possible that the mouse sequence is more susceptible. It is also possible, given the unnatural differentiation process for iPSC-derived cells in vitro, that different outcomes might be obtained in native tissues, and additional studies will be necessary to confirm whether or not such splicing occurs in R1154 mutant human CS. Nevertheless, the finding that R1154Q induces such splicing in any genome illustrates the principle that SUR2 GOF mutations can also be associated with additional LOF resulting from variable splicing that leads to reduced protein levels, such that the net effect could be either GOF or LOF in different tissues. We have demonstrated that isolated SUR2 LOF results in a very distinct constellation of features in ABCC9-related intellectual myopathy syndrome (AIMS) (49). Hence, dual GOF/LOF consequences of CS mutations could result in not just quantitatively, but qualitatively variable outcomes and marked variability of CS pathologies, and hence might underlie the marked variation in severity of CS consequences that is seen in patients (24).

### Conclusions.

Recent studies have defined the genetic basis of CS — first recognized as a distinct syndrome 30 years ago — as GOF in K_ATP_ channel genes, and have further defined the consequent mechanistic basis of multiple CS features. The most common human CS mutations, SUR2[R1154Q] and [R1154W], are present in approximately 30% of patients with CS. In the present study, we have shown that when introduced into the mouse locus, the SUR2[R1154Q] equivalent mutation caused canonical features of CS, but also the unanticipated consequence of alternate mRNA splicing, which resulted in a decrease in functional SUR2 protein levels. This is effectively a LOF that counteracts the mutational GOF action and leads to lower CS phenotypic severity. While studies in cells from SUR2 R1154Q and R1154W patient cells failed to reveal a similar outcome, the possibility remains that these or other GOF CS mutations might result in a counteracting loss of functional protein levels by a similar mechanism, which could then help explain, and have significant implications for, the wide variability of CS disease expressivity.

## Methods

### CRISPR/Cas9 genome editing

Using CRISPR/Cas9-mediated genome engineering technology (50), we generated knockin mice carrying a human GOF mutation in the *ABCC9* gene, which encodes the accessory SUR2 subunit of the K_ATP_ channel. Guide RNA (gRNA) target sequences predicted using the MIT CRISPR design tool (http://crispr.mit.edu) were cloned into plasmid pX330 (Addgene 42230). sgRNA activity was validated in vitro by transfection of N2A cells using Roche X-tremeGENE HP (MilliporeSigma), followed by T7E1 assay (New England BioLabs Inc.). The T7 sgRNA template and T7 Cas9 template were prepared by PCR amplification and gel purification, followed by RNA in vitro transcription with the MEGAshortscript T7 kit (gRNA) or the T7 mMessage mMachine Ultra kit (Cas9). After transcription, RNA was purified with the Megaclear kit (Life Technologies). 200 nt ssODN donor DNAs with the appropriate mutation centered within the oligonucleotide were synthesized by Integrated DNA Technologies as ultramer oligonucleotides.

B6CBA F1/J female mice (3–4 weeks old; The Jackson Laboratory) were superovulated and mated overnight with B6CBA F1/J male mice (>7 weeks old). Zygotes were harvested from the ampullae of superovulated females and placed in potassium-supplemented simplex optimized medium (KSOM; MR106D) before microinjection. Microinjection of the Cas9, sgRNA, and ssDNA template (at a final concentration of 50 ng/μL Cas9 WT RNA, 25 ng/μL gRNA, and 20 ng/μL ssODN DNA) was performed in flushing holding medium (FHM; EmbryoMax, MR-024-D, MilliporeSigma). After injection, zygotes were incubated at 5.5% CO_2_ at 37°C for 2 hours, and surviving embryos were transferred to ICR recipient mice (The Jackson Laboratory) by oviduct transfer. Founders were identified using a QIAGEN pyrosequencer and Pyromark Q96 2.5.7 software. We identified multiple viable and fertile positive founder mice carrying the SUR2[R1151Q] mutation (equivalent to human SUR2[R1154Q]), and we refer to these as SUR2[R1154Q] mice for direct comparison to the human CS equivalent. Successful mutation was verified in founder (F_0_) mice by Sanger sequencing of gDNA. Mutant mice were subsequently crossed with C57BL/6J mice (The Jackson Laboratory) to generate heterozygous F_1_ SUR2^WT/RQ^ lines. PCR was used to generate amplicons of *ABCC9* spanning more than 5 kb on either side of the introduced mutation, from gDNA isolated from mouse tails, and resultant PCR products were sequenced to confirm the absence of additional, unintended mutations. After verification, 1 F_1_ animal from 1 line of each genotype was selected and subsequently bred with C57BL/6J mice for multiple (>6) generations to generate the hetero- and homogeneous R1154Q as well as WT littermates that were used in experiments.

### Generation of human iPSCs and analysis of derived cardiomyocytes

R1154W patient RECs were reprogrammed to hiPSCs by the Washington University School of Medicine in St. Louis Genome Engineering and iPSC Core (GEiC) using Sendai virus–based reprogramming vectors. After 4 unsuccessful attempts to reprogram R1154Q patient RECs, PBMCs were provided by the patient, and were successfully reprogrammed by the GEiC using the Sendai virus–based reprogramming cocktail. hiPSCs were maintained on a 4-day passaging cycle. Differentiation to cardiomyocytes was carried out in entirely chemically defined conditions via temporal modulation of canonical Wnt signaling (33).

### RNA extraction and analysis

RNA was isolated from freshly dissecting cardiac apices or from iPSC-derived cardiomyocyte cultures using TRIzol (Thermo Fisher Scientific), and first-strand cDNA was synthesized using SuperScript III First-Strand Synthesis System (Thermo Fisher Scientific).

### Protein analysis

#### Protein extraction from heart tissue.

Snap-frozen tissue was thawed on ice and equilibrated with ice-cold homogenization buffer (protease inhibitors, 50 mM NaCl, 0.32 M sucrose, 2 mM EDTA, 20 mM HEPES pH 7.4). Atria were dissected from ventricles. The ventricular tissue was diced, resuspended in homogenization buffer, and homogenized via a Miccra D-1 homogenizer and subsequent strokes by a manual glass-Teflon Dounce homogenizer. The suspension was then centrifuged at 100,000*g*. The obtained membrane pellet was resuspended in homogenization buffer, aliquoted, and snap frozen with liquid nitrogen. Membranes were resuspended in solubilization buffer (1.5% Triton X-100, 0.75% sodium deoxycholate, 0.1% SDS, protease inhibitors in 10 mM NaCl, 5 mM EDTA, 2.5 mM EGTA, 50 mM Tris-HCl pH 7.35) and centrifuged at 50,000*g* at 4°C. Supernatant was subjected to TCA to a final concentration of 12.5 % and incubated for 30 minutes on ice. The pellet was acetone washed twice and air dried at 37°C; supplemented with 1× SDS sample buffer (50 mM Tris-HCl pH 6.8, 2% SDS, 0.1% bromophenol blue, 10% glycerol) containing 100 mM DTT; and resuspended for subsequent analysis by SDS-PAGE.

#### Glycosidase treatment.

40 μL glycoprotein denaturing buffer (reconstituted with 1× glycoprotein denaturing buffer, 2.5% NP-40, 1× G7 in ddH_2_0) was added to TCA-precipitated air-dried pellets from approximately 100 μg total protein and agitated at room temperature for 30 minutes. Subsequently, 1.5 μL of the glycosidase (PNGase F, 750 U; New England BioLabs Inc.) was added to the mixture. After incubation at 37°C for 1 hour at 1000 rpm, the mixture was supplemented with 5× SDS sample buffer and 100 mM DTT, agitated for 30 minutes, and analyzed by SDS-PAGE.

#### Protein analysis by Western blotting.

For separating proteins via SDS-PAGE, 6% polyacrylamide gels were used for proteins greater than 100 kDa and 12% for other proteins. Electrophoresis was performed at constant current, limited to 15 mA per gel. Gels with separated proteins were put onto a nitrocellulose membrane and placed between 2 blotting papers, and electroblotted for 90 minutes in transfer buffer (25 mM Tris, 192 mM glycine, pH 8.3) at 4°C with a constant voltage of 60 V and the current limited to 1 A. Membranes were washed and blocked with blocking buffer (5% wt/vol milk powder, 25 mM Tris/HCl pH 7.4, 135 mM NaCl, 3 mM KCl, 0.02% IGEPAL).

As previously described, the anti-Kir6.2 antibody (raised in guinea pig and yielded as serum of the third bleeding; ref. 51) recognizes the last 36 amino acids of the protein and was characterized on native tissue against Kir6.2-knockout controls (28). Information about antibodies against proteins other than Kir6.2 is shown in Table 1. Primary antibodies were diluted in blocking buffer and incubated overnight at 4°C. For antibodies against SUR proteins, a different blocking buffer (“SUR-blocking buffer”: 4% wt/vol milk powder, 25 mM Tris/HCl pH 7.4, 135 mM NaCl, 3 mM KCl, 0.1% Tween-20) was used. Subsequently, membranes were washed 3 times with their respective blocking buffer and incubated with IRDye LI-COR secondary antibodies (800CW) diluted in blocking buffer at 1:4000. Blots were incubated for 90 minutes at room temperature and washed with washing buffer (25 mM Tris/HCl pH 7.4, 135 mM NaCl, 3 mM KCl, 0.1% Tween-20 for SUR proteins, 5% wt/vol milk powder, 25 mM Tris/HCl pH 7.4, 135 mM NaCl, 3 mM KCl, 0.02% IGEPAL for others), and antibody signals were subsequently visualized using an Odyssey Sa Infrared imaging system.

### Patch clamp electrophysiology

#### Isolated VSMCs.

Mice were anesthetized with 2.5% avertin (10 mL/kg, i.p.; MilliporeSigma), and the ascending aorta was rapidly dissected and placed in ice-cold physiological saline solution (PSS) containing (in mM): NaCl 134, KCl 6, CaCl_2_ 2, MgCl_2_ 1, HEPES 10, and glucose 10, with pH adjusted to 7.4 with NaOH. Smooth muscle cells were enzymatically dissociated in dissociation solution containing (in mM): NaCl 55, sodium glutamate 80, KCl 5.6, MgCl_2_ 2, HEPES 10, and glucose 10, pH 7.3 with NaOH, then placed into dissociation solution containing papain 12.5 μg/mL, DTT 1 mg/mL, and BSA 1 mg/mL for 25 minutes (at 37°C), before immediate transfer to dissociation solution containing collagenase (type H:F = 1:2) 1 mg/mL and BSA 1 mg/mL for 5 minutes (at 37°C). Cells were dispersed by gentle trituration using a Pasteur pipette, plated onto glass coverslips on ice and allowed to adhere for more than 1 hour before transfer to the recording chamber.

Whole-cell K_ATP_ currents were recorded using an Axopatch 200B amplifier and Digidata 1200 (Molecular Devices). Recordings were sampled at 3 kHz and filtered at 1 KHz. Currents were initially measured at a holding potential of –70mV in high-Na^+^ bath solution containing (in mM): NaCl 136, KCl 6, CaCl_2_ 2, MgCl_2_ 1, HEPES 10, and glucose 10, with pH adjusted to 7.4 with NaOH before switching to a high-K^+^ bath solution (KCl 140, CaCl_2_ 2, MgCl_2_ 1, HEPES 10, and glucose 10, with pH adjusted to 7.4 with KOH) in the absence and presence of pinacidil and glibenclamide as indicated. The pipette solution contained (in mM) potassium aspartate 110, KCl 30, NaCl 10, MgCl_2_ 1, HEPES 10, CaCl_2_ 0.5, K_2_HPO_4_ 4, and EGTA 5, with pH adjusted to 7.2 with KOH.

#### Isolated ventricular myocytes.

Ventricular myocytes were isolated from adult mice, anesthetized using 2.5% Avertin (10 mL/kg), and the heart and ascending aorta were removed and immersed in ice-cold calcium free Wittenberg isolation medium (WIM; in mM): 116 NaCl, 5.4 KCl, 8 MgCl_2_, 1 NaH_2_PO_4_, 1.5 KH_2_PO_4_, 4 NaHCO_3_, 12 glucose, 21 HEPES, 2 glutamine plus essential vitamins (Gibco) and essential amino acids (Gibco) (pH 7.40). The heart was cannulated via the aorta and Langendorff perfused with WIM for 5 minutes at 37°C, followed by 20 minutes of perfusion with WIM supplemented with 270 U/ml collagenase type 2 (Worthington Biochemical Corp.) and 10 μM CaCl_2_ at 37°C. The heart was then transferred to WIM containing 50 mg/mL BSA, 12.5 mg/mL taurine, and 150 μM CaCl_2_; and ventricular tissue was manually dissociated using forceps before single-cell dissociation by trituration with a fire-polished Pasteur pipette.

Inside-out patch clamp recordings were made in symmetrical KINT solution which contained (in mM): 140 KCl, 10 HEPES, 1 EGTA (pH 7.4 with KOH). Varying MgATP concentrations were applied using a Dynaflow Resolve perfusion chip (Cellectricon). MgCl_2_ was added to each solution to achieve a free [Mg^2+^] 0.5 mM according to calculations using CaBuf (Katholieke Universiteit Leuven). Membrane currents were sampled at 3 KHz and filtered at 1 KHz at a holding potential of –50 mV using an Axopatch 700B amplifier and Digidata 1200 (Molecular Devices). K_ATP_ channel currents in solutions of varying nucleotide concentrations were normalized to the basal current in the absence of nucleotides, and dose-response data were fit with a 4-parameter Hill fit according to the following equation: *Normalized current* = *I* + (*I_max_* – *I_min_*)/(1 + ([*X*]/IC_50_)*^H^*); where the current in *K_int_* = *I_max_* = 1, *I_min_* is the normalized minimum current observed in MgATP, [*X*] refers to the concentration of MgATP, *IC_50_* is the concentration of half-maximal inhibition, and *H* denotes the Hill coefficient.

Whole-cell patch clamp recordings of voltage-gated calcium channel activity were made in a bath solution that contained (in mM): 116 NaCl, 5.4 CsCl, 0.16 NaH_2_PO_4_, 10 glucose, 1.8 CaCl_2_, 0.5 MgCl_2_, 5 HEPES, 3 NaHCO_3_, and 0.01 tetrodotoxin (pH 7.4 with NaOH) using a pipette solution that contained (in mM): 120 CsCl, 20 TEA-Cl, 5 K_2_ATP, and 10 HEPES (pH 7.3 with KOH). Cell capacitance and series resistance were determined from 5 mV square pulses from a holding potential of –70 mV following establishment of the whole-cell configuration. All recordings were performed at 20°C–22°C.

### Arterial compliance

After mice were euthanized under isoflurane anesthesia, the ascending aorta and left common carotid artery of 3-week-old mice were excised and placed in a PSS containing 130 mM NaCl, 4.7 mM KCl, 1.18 mM MgSO_4_-7H_2_O, 1.17 mM KH_2_PO4, 14.8 mM NaHCO_3_, 5.5 mM dextrose, and 0.026 mM EDTA (pH 7.4). The vessels were then cleaned from surrounding fat, mounted on a pressure arteriograph (Danish Myo Technology), and maintained in PSS at 37°C. Vessels were visualized with an inverted microscope connected to a charge-coupled device camera and a computerized system, which allowed continuous recording of vessel diameter. Intravascular pressure was increased from 0 to 175 mmHg by 25 mmHg increments, and the vessel outer diameter was recorded at each step (12 seconds per step). The average of 3 measurements at each pressure was reported.

### BP measurement

#### In anesthetized mice.

Mice were anesthetized with 1.5% inhaled isoflurane and restrained on a heating pad to maintain body temperature. A 2- to 3-mm incision was made in the midline of the neck; the thymus and muscle were separated to expose the right carotid artery. A Millar pressure transducer (model SPR-671) was inserted into the right carotid artery and moved to the ascending aorta. Systolic BP (SBP), diastolic BP (DBP), and HR were recorded using the PowerLab data acquisition system (ADInstruments), and data were analyzed using LabChart 7 (ADInstruments). For blood pressure measurements in conscious mice, a radio-telemetry pressure transmitter (DSI) was surgically inserted into the left carotid artery and moved to the ascending aorta, where BPs during day and night were recorded by the DSI data acquisition system after mice recovered from surgery.

#### Telemetry probe implantation and telemetry recording.

Mice (6–8 months old) were implanted with TA11PA-C10 (DSI) telemetric implants under anesthesia, with a gas concentration of 1.5%–2.5% isoflurane. The catheter was advanced into the ascending aorta via the left carotid artery, and the body of transmitter was slipped into the pocket subcutaneously in the right flank. Animals were housed in an isolated recording room and allowed at least 1 week of recovery before recordings were taken. Systolic (SBP), diastolic (DBP), mean arterial pressure (MBP = DBP + 1/3[SBP — DBP]), and HR were collected using the Dataquest ART system. Data were sampled by averaging 10 seconds of each 1-minute period. Values of day and night were averages of day time (6 am–6 pm) or night time (6 pm–6 am). After 3 days of baseline recording, the mice were injected with pinacidil (i.p. 0.01, 0.1, 1 mg) daily.

### Heart weight measurement and histology

Mice were anesthetized with 2.5 % Avertin, and hearts were excised and rinsed with PBS, which contained (in mM): 137 NaCl, 2.7 KCl, 10 Na_2_HPO_4_, KH_2_PO_4_ (pH 7.4 with NaOH). The hearts were arrested in diastole with 10% KCl and blotted to remove excess liquid. Hearts were then weighed, and weight was normalized to tibia length. After weighing, the hearts were fixed in 10% buffered formalin for 24 hours and embedded in paraffin. Sections (3 μm) were cut and stained with H&E for the morphometric analysis.

### Echocardiography

Short-axis left ventricular scans were obtained via M-mode echocardiography using an ATL 5000cv instrument (Phillips) with a 15-MHz compact linear array. The operator was blinded to genotype. Left ventricular end-diastolic dimension (LVEDD), LV end-systolic dimension (LVESD), end diastolic anterior wall thickness (AWT), end diastolic posterior wall thickness (PWT), R-R interval, and ejection time (ET) were recorded from 3 separate cardiac cycles for each mouse. Wall thickness divided by chamber radius was calculated at diastole. LV mass (LVM) was calculated using the Devereux equation. Fractional shortening (FS%) refers to (LVEDD – LVESD)/LVEDD as a percentage. Stroke volume (SV) refers to the amount of blood ejected by the left ventricle in one contraction, determined by subtracting LV end-systolic volume from LV end-diastolic volume (LVEDV – LVESV), assuming LVEDV and LVESV are simply cubed. The ejection fraction (EF%; SV/LVEDV) refers to the percentage of blood that is pumped out of the ventricles with each contraction.

### Statistics

Unless otherwise noted, all data are presented as mean ± SEM and were tested for statistical significance using 1-way ANOVA, with post hoc Tukey’s test or 2-tailed Student’s *t* test as indicated. *P* values less than 0.05 were considered statistically significant.

### Study approval

Studies were performed in compliance with the standards for the care and use of animal subjects defined in the *Guide for the Care and Use of Laboratory Animals* (National Academies Press, 2011) and were reviewed and approved by the Washington University Institutional Animal Care and Use Committee. All human studies were approved by the Washington University Human Studies Committee and carried out with the full written consent of participating patients.

## Author contributions

All authors made equally significant contributions to this study: HZ, MSR, and CGN originally conceived the study; MSR and CGN oversaw the generation of the mutant mice; HZ, AH, TSdA, CM, CE, ECA, TH, ZY, PEC, GSB, AK, and CMH carried out the experiments; RPM, GKS, DKG, and BS contributed key technical help or clinical background; HZ, AH, CM, BS, MSR, and CGN wrote the manuscript, which was edited by the other authors.

## Figures and Tables

**Table 1:**
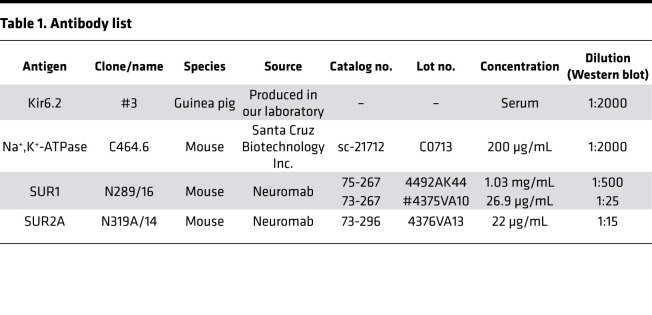
Antibody list

## Version 1 02/02/2021

In-Press Preview

## Version 2 03/08/2021

Electronic publication
